# What is the significance of onconeural antibodies for psychiatric symptomatology? A systematic review

**DOI:** 10.1186/s12888-017-1325-z

**Published:** 2017-05-03

**Authors:** Sverre Georg Sæther, Morten Schou, Daniel Kondziella

**Affiliations:** 1 Department of Psychiatry, St. Olav’s University Hospital, Pb. 3008, Lade, 7441 Trondheim, Norway; 2 Department of Mental Health, Norwegian University of Science and Technology, Faculty of Medicine and Health Science, Pb. 8905, 7491 Trondheim, Norway; 3 Department of Neurology, Rigshospitalet, Copenhagen University Hospital, Blegdamsvei 9; DK, -2100 Copenhagen, Denmark

**Keywords:** Paraneoplastic neuro-psychiatric syndromes, Onconeural antibodies, Anti-neuronal antibodies, Review

## Abstract

**Background:**

Patients with intracellular onconeural antibodies may present with neuro-psychiatric syndromes. We aimed to evaluate the evidence for an association between well-characterized onconeural antibodies and psychiatric symptoms in patients with and without paraneoplastic central nervous system syndromes.

**Methods:**

Eligible studies were selected from 1980 until February 2017 according to standardized review criteria and evaluated using Quality Assessment of Diagnostic Accuracy Studies−2 (QUADAS-2). We included studies describing the psychiatric symptomatology of onconeural antibody positive patients and the prevalence of onconeural antibodies in patients with psychiatric disorders.

**Results:**

Twenty-seven studies met the inclusion criteria. Six studies reported on the prevalence of well-characterized onconeural antibodies in patients with different psychiatric disorders, ranging from 0% to 4.9%. Antibody prevalence in controls was available from three studies, ranging from 0% to 2.8%. Data heterogeneity precluded a meta-analysis. Two cerebrospinal fluid studies found well-characterized onconeural antibodies in 3.5% and 0% of patients with psychotic and depressive syndromes, respectively.

**Conclusions:**

The available evidence suggests that the prevalence of well-characterized onconeural antibodies in patients with psychiatric disorders is generally low. However, the question whether onconeural antibodies are important in select patients with a purely psychiatric phenotype needs to be addressed by appropriately designed studies in the future.

**Electronic supplementary material:**

The online version of this article (doi:10.1186/s12888-017-1325-z) contains supplementary material, which is available to authorized users.

## Background

Paraneoplastic central nervous system (CNS) syndromes can be defined as remote effects of cancer on the brain that are not caused by tumor infiltration, metastases, metabolic or nutritional deficits, secondary infections or oncological treatment [1]. These syndromes are strongly associated to well-characterized onconeural antibodies and present with psychiatric and/or neurological symptoms [2–4]. For instance, paraneoplastic limbic encephalitis typically evolves over days to weeks and includes memory disturbances and seizures, as well as psychiatric symptoms such as irritability, hallucinations, depression, and disturbances of mood and personality [2, 5]. Onconeural antibodies target intracellular antigens in tumors and neuroectodermal tissues and are associated with various types of cancer and clinical syndromes (See Table 1 for details) [6, 7]. Well-characterized onconeural antibodies include anti-Hu (ANNA-1), -Ri (ANNA-2), -Yo, -CRMP5 (CV2), -Ma1, -Ma2 (Ta), -Amphiphysin, -Recoverin, -Tr and -SOX1 [7, 8]. In contrast to the well-documented pathogenicity of antibodies targeting neuronal surface antigens (e.g. anti-NMDAR), it is assumed that onconeural antibodies represent an epiphenomenon to cytotoxic T-cell reactions [9, 10]. There is, however, some evidence for their direct pathogenicity. For instance, one group of researchers recently found anti-Yo to cause dysregulation of the calcium homeostasis in Purkinje cells in rat cerebellar slice cultures [11]. A different research group demonstrated that anti-Hu and anti-Yo induce neuronal and Purkinje cell death, respectively, in hippocampal and cerebellar slice cultures from rats [12–14].

**Table 1:** Well-characterized onconeural antibodies and their associated tumors and syndromes (Modified after [7])

Onconeural antibody	Tumors^a^	CNS syndromes^a^
Hu (ANNA-1)	SCLC, other lung ca., prostate ca.	PCD, LE, PEM, OMS, BE, myelitis
Ri (ANNA-2)	Mamma ca., SCLC, other lung ca.	BE, OMS, PCD, LE, PEM, myelitis
Yo (PCA-1)	Ovary ca., uterus ca., mamma ca.	PCD
CRMP-5 (CV2)	SCLC, other lung ca., thymoma	PCD, LE, PEM, BE, myelitis
Ma1	Lung ca., mamma ca., colon ca.	BE, PCD, PEM, LE, OMS
Ma2 (Ta)	Testis ca.	LE, PCD, Dienc. and brainstem symptoms
Amphiphysin	Mamma ca., SCLC, colon ca.	SPS, PEM, PCD, LE
Recoverin	SCLC, other lung ca.,mamma ca.	Retinopathy
Tr	Lymphoma	PCD
SOX1	SCLC	LE, PCD, BE

^a^Only most frequent tumors and syndromes described. Abbreviations: BE Brainstem encephalitis, ca cancer CNS Central nervous system, Dienc Diencephalic, LE Limbic encephalitis, OMS Opsoclonus-myoclonus-syndrome, PCD Paraneoplastic cerebellar degeneration, PEM Paraneoplastic encephalomyelitis, SCLC Small cell lung cancer, SPS Stiff-person-syndrome

The psychiatric literature on onconeural antibodies and paraneoplastic neurological syndromes is sparse [15]. Some authors have suggested that onconeural antibodies may play a role as a marker of autoimmune processes in subgroups of patients with psychiatric disorders [16, 17]. If this is true, onconeural antibody status might have implications for choice of therapeutic strategy and possibly also indicate the need for tumor screening in serum positive patients. On the other hand, if the hypothesis is false, onconeural antibody testing may be unnecessary.

We aimed to determine the evidence for an association of onconeural antibodies with the occurrence, persistence or worsening of psychiatric symptoms in patients with (and without) paraneoplastic CNS disease. Using the PICO approach [18], we phrased the following primary research question: In patients with psychiatric symptoms (Population), does a positive onconeural antibody test result (Intervention) compared to a negative test result (Comparison) predict a different psychopathological profile, i.e. greater burden of affective, cognitive and/or psychotic symptoms (Outcome)? For secondary outcomes: Do patients with 1) malignancies or 2) paraneoplastic syndromes (P) and a positive onconeural antibody test result (I), as compared to those with a negative test result (C), present with a different psychopathological profile (O)?

## Methods

We performed a systematic review using standardized methods (Quality Assessment of Diagnostic Accuracy Studies-2 (QUADAS-2) and Preferred Reporting Items for Systematic Reviews and Meta-analyses (PRISMA) [19, 20]). The review protocol was registered in the PROSPERO database (registration number CRD42015025826) and can also be accessed from Additional file 1.

### Criteria for considering studies for this review

#### Types of studies

The criteria for inclusion and exclusion of studies are presented in Table 2.

**Table 2:** Inclusion and exclusion criteria for the systematic review

Inclusion criteria	Aims: * Prevalence of well-characterized onconeural antibodies in patients with psychiatric disorders * Psychiatric symptomatology of onconeural antibody positive patients in patients with psychiatric disorders, malignancies and/or paraneoplastic neurological syndromes Design: Retrospective, prospective, observational and/or interventional
Exclusion criteria	Study sample < 5 patients Pediatric or adolescent patients (age < 18) Non-validated methods for onconeural antibody testing

#### Index test and reference standards

The index test comprised onconeural antibodies in patient serum or cerebrospinal fluid. We addressed the following well-characterized onconeural antibodies: anti-Hu (ANNA- 1), -Ri (ANNA-2), -Yo, -CRMP5 (CV2), -Ma1, -Ma2 (Ta), -Amphiphysin, -Recoverin, -Tr and -SOX1. Established clinical criteria for malignancies, paraneoplastic neurological syndromes and psychiatric disorders according to standard clinical criteria, e.g. ICD-10, DSM-III-R, DSM-IV and DSM-IV-TR were considered as reference standards [21–24].

### Search methods for identification of studies

We searched the following databases for relevant literature from 1 January 1980 to 15 February 2017: Cochrane Central Register of Controlled Trials (The Cochrane Library), Medline (PubMed), EMBASE, and clinicaltrials.gov. The search was based on the following terms: Paraneoplastic syndromes, onconeural antibodies, psychiatry, mental disorders, depression, psychosis, neoplasms, cancer, antibodies. See Additional file 1 for details. An academic librarian supervised the literature search. We manually searched the references from relevant manuscripts to identify additional articles. Further, we cross-referenced the papers using the “cited by” function on PubMed. Non-English literature were included if an English Abstract was available and a reliable translation of the manuscript into English possible. If necessary, personal communication with authors was attempted via email in order to obtain additional data.

### Data collection, analysis, and reporting

#### Selection of studies, data extraction, and management

In papers with relevant titles, abstracts were evaluated. Eligible studies were then identified on the basis of their full text. One author (SGS) performed the initial selection, whereas two authors (SGS and MBS) performed a quality assessment. One of the authors (SGS) extracted the relevant information from each study, which was validated by a second author (MBS).

#### Assessment of methodological quality, including investigations of heterogeneity

Two of the authors (SGS and MBS) independently assessed the methodological quality of each included study using the Quality Assessment of Diagnostic Accuracy Studies-2 (QUADAS-2) [19]. The QUADAS-2 has four domains: (1) participant selection, (2) index test, (3) reference standard, and (4) flow of participants through the study and timing of the index tests and reference standard (flow and timing). Risk of bias is judged as “low”, “high” or “unclear” for each domain. The first three domains are also assessed for concerns regarding applicability. A third author (DK) resolved disagreement if consensus could not be reached by the two reviewing authors.

#### Statistical analysis, data synthesis, and reporting

We aimed to perform a meta-analysis of the available numerical data reporting on 1) the frequency of onconeural antibodies in patients with psychiatric symptoms and/or diagnoses, 2) the frequency of psychiatric symptoms in patients with onconeural antibodies, and 3) the psychopathological profile of patients with onconeural antibodies. However, a meta-analysis was judged as meaningful only if design and quality of the included studies were deemed satisfactory. Data were reported according to the PRISMA criteria [20] (Additional file 1).

## Results

Results are summarized in Tables 3 and 4 and Fig. 2 **.**

**Table 3:** Systematic evaluation of publications included in the review using Quality Assessment of Diagnostic Accuracy Studies-2 [19]

Study	Risk Of Bias				Applicability Concerns		
	Patient selection	Index test	Reference standard	Flow and timing	Patient selection	Index test	Reference standard
Hammack et al. 1990 [25]	Unclear	Low	High	High	High	High	High
Dalmau et al. 1992 [26]	Low	Low	High	High	High	Low	High
Peterson et al. 1992 [27]	Low	Low	High	High	High	Low	High
Alamowitch et al. 1997 [28]	Low	Low	High	High	High	Low	High
Black et al. 1998 [29]	Unclear	Low	Low	Low	Low	Low	Low
Voltz et al. 1999 [31]	Low	Low	High	High	High	Low	High
Antoine et al. 1999 [30]	Low	Low	High	High	High	Low	High
Gultekin et al. 2000 [2]	Unclear	Low	High	High	High	Low	High
Yu et al. 2001 [32]	Low	Low	High	High	High	Low	High
Sillevis Smitt et al. 2002 [33]	Low	Low	High	High	High	Low	High
Lawn et al. 2003 [34]	High	Low	High	High	High	Low	High
Overeem et al. 2004 [36]	High	Low	High	High	High	Low	High
Dalmau et al. 2004 [35]	Low	Low	High	High	High	Low	High
Hoffmann et al. 2008 [37]	Unclear	Low	High	High	High	Low	High
McKeon et al. 2011 [38]	Low	Low	High	High	High	Low	High
Chiaie et al. 2012 [39]	High	Low	Low	Low	Low	High	Low
Saraya et al. 2013 [40]	Low	Low	High	High	High	Low	High
Dahm et al. 2014 [41]	Unclear	Low	Low	Low	Low	Low	Low
Moon et al. 2014 [47]	Low	Low	High	High	High	Low	High
Laadhar et al. 2015 [17]	Low	Low	Low	Low	Low	Low	Low
Haukanes et al. 2015 [43]	High	Low	Low	Low	Low	Low	Low
Kruse et al. 2015 [44]	High	Low	Unclear	Low	Low	Low	Unclear
Endres et al. 2015 [42]	High	Low	Unclear	Low	Low	Low	Unclear
Sæther et al. 2016 [46]	Low	Low	Low	Low	Low	Low	Low
Endres et al. 2016 [45]	High	Low	Unclear	Low	Low	Low	Unclear
Hansen et al. 2016 [48]	High	Low	High	High	High	Low	High
Schwenkenbecher et al. 2016 [49]	Low	Low	High	High	High	Low	High

**Table 4:** Results from a systematic review of studies included in the review

Source	Country-period	Sites (design)	Study population	Nr. of individuals with IgG onc. Abs./ in study	Patients with tumors	Age (y, median/ *mean); % female	Abs studied	Onc. Abs. studied	Analytic method	Psychiatric reference standard (ICD-10, DSM-IV and IIIR)	Prevalence psychiatric symptoms in study/onc. Ab. pos. Pat.	Psychiatric symptoms reported in onc. Ab. pos. Pat.
Hammack et al. 1990 [25]	US (NY) -?	S (r)	PCD	16/32	27/32	?; 50	Anti-Purk. cell Abs	Anti-Purk. cell Abs	IIF	None	28%/38%	Depr., psychosis
Dalmau et al. 1992 [26]	US (NY) – 83-90	3 (r)	Hu-ass par. enc. myel./ sens neur.	71/71	62/71	60; 55	Hu	Hu	WB, Im.hi.	None	21%/21%	Depr., anxiety, mem. Loss, hall.
Peterson et al. 1992 [27]	US -? -91	M (r)	Yo-pos pat	55/55	52/55	61; 100	Yo	Yo	WB, Im.hi.	None	18%/18%	Emo. lab., mem. Def.
Alamowitch et al. 1997 [28]	? - 87-94	S (r)	LE and SCLC	8/16	16/16	?; 13	Hu	Hu	Im.bl, Im.hi.	None	88%/88%	Depr., anx., pers. ch, hall.
Black et al. 1998 [29]	US (Roch) -?	S (c)	OCD pat	0/13	?	36; 46	14	Yo, Hu, Ri, Amph	IIF	DSM-IIIR	100%/−	-
Voltz et al. 1999 [31]	US (NY) -?	S (r)	Testis cancer and PLE	10/19	19/19	?; 0	Ma2	Ma2	WB, Im.hi.	None	?/40%	Anxiexy., depr., hall.
Antoine et al. 1999 [30]	France - 72-96	1 (r)	Amphiphysin pos pa	5/5	5/5	67; 40	Amph.	Amph.	WB, Im.hi.	None	?/20%	Anxiexy., depr., hall.
Gultekin et al. 2000 [2]	US (NY) -?	S (r)	PLE	30/50	48/50	55; 46	6	Hu, Ma1, Ma2, CRMP-5/CV2	Im.bl, Im.hi.	None	42%/?	?
Yu et al. 2001 [32]	US (Minn) - 93-00	1 (r)	CRMP5 (CV2) pos patients	116/116	105/116	?; 58	CRMP-5/CV2	CRMP-5/CV2	Im.bl, IIF	None	21%/21%	Pers ch., depr., psychosis
Sillevis Smitt et al. 2002 [33]	Netherlands– 89 – 99	S (r)	Hu-ass. par. enc.myel./ sensory neuronopathy	73/73	62/73	66; 34	Hu	Hu	WB, IIF	None	2.7%/2.7%	Depr.
Lawn et al. 2003 [34]	Us (Roch) 85–02	S (r)	PLE	10/24	24/24	61; 63	11	Hu, Ri, Yo, Tr, CRMP-5/CV2, Amph	WB, IIF	None	50/?	Depr., anxiety, pers. ch, hall.
Overeem et al. 2004 [36]	US (AR) -?	S (r)	Ma2 ass. enc.	6/6	6/6	49; 50	Ma2	Ma2	Im. bl.	None	50%/50%	Depr., pers. ch.
Dalmau et al. 2004 [35]	US (Penn) -?	2 (r)	Ma2-ass. enc.	38/38	34/38	?; 32	Ma1/Ma2	Ma1/Ma2	Im. bl.	None	11%/11%	Nerv. br.down, loss of self-conf., panic attack
Hoffman et al. 2008 [37]	Germany/England – 99-05	2 (r)	Anti-Ma1/Ma2 ass. PNS	22/22	17/22	60; 36	Ma1/Ma2	Ma1/Ma2	Im.bl., IIF.	None	18%	OCS, pers. ch., aff. Symptoms
McKeon et al. 2011 [38]	US (Minn, Ari, Flor) - 87-07	3 (r)	Yo-pos p	83/83	73/83	60; 100	Yo	Yo	IIF	None	5%/5%	Pers. ch.
Chiaie et al. 2012 [39]	Italy -?	S (c)	Psychiatric pat PCD pat Healthy controls	11/48 22/22 0/52	0/48 22/22 0/52	36*; 13 56*; 68 44*; 50	5	Anti-Purk. cell Abs	IIF	DSM-IV	100%/100%	Schizophrenia, bipolar disord., OCD
Saraya et al. 2013 [40]	Thailand. - 10-12	17 (p)	Autoimmune encephalitis	9/103	6/103	?; 68	20	Hu, Ri, Yo, Tr, CRMP-5/CV2, Amph, SOX1	Im.bl, IIF	None	?/33%	Psychosis, Beh ch.
Dahm et al. 2014 [41]	Germany – 05-11	M (c)	Healthy Schizophrenia Aff. disord. Bord. pers. disord.	47/1703 38/1378 8/310 0/42	?	?; 40	24	Amph, Ma1, Ma2, CRMP-5/CV2, Tr, Hu, Rec, Ri, Yo	IIF	DSM-IV	100%/100%	-
Moon et al. 2014 [47]	South Korea– 12-14	S (r)	Non-stiff amphiphysin syndrome	20/20	7/20	58*; 40	12	Amph., Hu, Yo, Ri, Ma2, CRMP-5.	Im.bl.	None	25%/25%	Irritability, psychosis
Laadhar et al. 2015 [17]	Tunisia -?	S (c)	Psychiatric pat Healthy controls	5/103 0/41	0/103 0/41	43*; 28 41*; 22	ANNA, PCA1, ANA	Hu, Ri, Yo	WB, IIF	DSM-IV	100%/100%	Schizophrenia, schizoaff. And bipolar disord.
Haukanes et al. 2015 [43]	Norway -?	S (c)	ADHD pat Healthy controls	0/169 0/56	0/169 0/56	33*; 50 35*; 55	Yo, Amph, CRMP-5, Ma2, Ri, Hu	Yo, Amph, CRMP-5/CV2, Ma2, Ri, Hu	Im.bl, WB, IIF	DSM-IV	100%/−	-
Kruse et al. 2015 [44]	US (Roch)- 02–11	S (r)	Psychiatric inpat Healthy controls	1/213 0/173	?	?;?	?	Hu, Ri, SOX1, Yo, CRMP-5/CV2, Amph, Tr	Im.bl, IIF, ELISA	Chart review	100%/100%	Pers ch, paranoia, agitation etc
Endres et al. 2015 [42]	Germany – 06-13	S (c)	Pat with psychotic synd. (CSF)	5/142 (180)	0/180	35*; 56	14	Yo, Hu, Ri, CRMP-5/CV2, Ma1, Ma2, SOX1, Amph	Im.bl.	?	100%/100%	Schizophreniform or schizoaff. synd.
Sæther et al. 2016 [46]	Norway – 04-06	S (c)	Acutely admitted psychiatric inpat	1/585	?	41*; 51.8	15	Yo, Amph, CRMP-5/CV2, Hu, Ri, Ma2, Rec	Im.bl, IIF	ICD-10	100%/100%	Paranoid psychosis
Endres et al. 2016 [45]	Germany – 06 – 13	S (c)	Pat with depressive synd. (CSF)	0/63 (125)	?	53*; 48	?	Yo, Hu, Ri, CRMP-5/CV2, Ma1, Ma2, SOX1, Amph	Im.bl.	?	100%/−	-
Hansen et al. 2016 [48]	Germany – 07-15	S (r)	PLE: GAD65 Onconeur. abs	0/11 11/11	? 1/11	41; 64 43; 73	22	Ma1/2, Hu, Ri, Yo, SOX1, CRMP-5, Recoverin, Amph.	Im. hi., Im.bl.		66%/73%	Depression
Schwenkenbecher et al. 2016 [49]	Germany – 96-15	S (r)	Hu-pos. Pat with PS	18/18	16/18	61; 61	Hu	Hu	Im.hi., im.bl.	None	?/?	Aggressive-ness, anxiety

*Abbreviations*: *Abs* Antibodies, *ADHD* Attention Deficit Hyperactivity Disorder, *Aff.* disord Affective disorder, *Amph* Amhiphysin, *Ari*. Arizona, *AR.* Arkansas, *Ass.* Associated, *Beh ch.* Behavior changes, *Bord. pers. disord.* Bordeline personality disorder, *C* Cross-sectional, *CRMP-5* Collapsin Response Mediator Protein-5, *CSF* Cerebrospinal Fluid, *Depr* Depression, *DSM* Diagnostic and Statistical Manual of Mental disorders, *ELISA* Enzyme-linked immunosorbent assay, *Emo. lab.* Emotional lability, *Enc.* Encephalitis, *Flor.* Florida, *Hall.* Hallucinations, *Hu-ass. par.enc.myel.* Hu-associated paraneoplastic encephalomyelitis, *ICD-10* International Classification of Diseases-10, *IIF* Indirect Immunofluorescence, *Im.bl.* Immunoblot, *Im. hi.* Immunohistochemistry, Inpat Inpatients, *LE* Limbic encephalitis, *Ma2-ass. enc.* Ma2-associated encephalitis, *M* Multicenter, *Mem.def.* Memory deficit, *Mem.* loss Memory loss, *Minn.* Minnesota, *Myel.* Myelitis, *Nerv.br.down* Nervous breakdown, *NY* New York, *OCD* Obsessive-compulsive disorder, *OCS* Obsessive-compulsive symptoms, *Onc.* Onconeural, *Pat* Patients, *P* Prospective, *PCD* Paraneoplastic Cerebellar Degeneration, *Pers ch.* Personality changes, *Pm ret.* Psychomotor retardation, *PCD* Paraneoplastic cerebellar degeneration, *Penn.* Pennsylvania, *PLE* Paraneoplastic Limbic Encephalitis, *PNS* Paraneoplastic neurological syndrome, *Pos.* Positive, *Purk.* cell Purkinje-cell, *Rec.* Recoverin, *R* Retrospective, *Roch.* Rochester, *S* Single-center, *Schizoaff. synd.* Schizoaffective syndrome, *SCLC* Small Cell Lung Cancer, *Self-conf.* Self-confidence, *Susp.* Suspected, *Synd.* Syndrome, *WB* Western Blot, *Y* Years

^*^Mean

### Systematic literature search and quality assessment

**Fig. 1:**
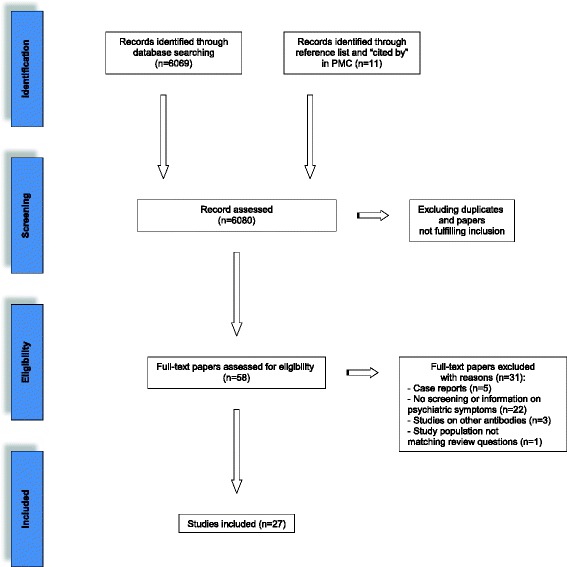
Schematic overview of the literature research

**Fig. 2:**
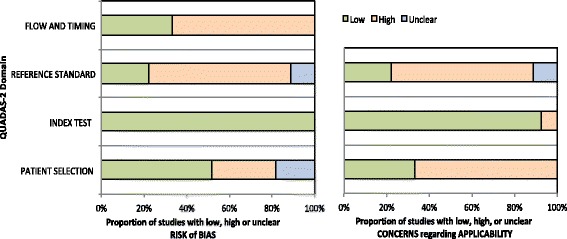
Systematic evaluation of bias and applicability concern in studies included in the review using QUADAS-2 [19]

The initial search yielded 6069 citations (Se Fig. 1 for Flow chart). We identified 27 original publications that met eligibility criteria [2, 17, 25–49]. Data about the serum prevalence of well-characterized onconeural antibodies in patients with psychiatric disorders were available from 6 papers [17, 29, 41, 43, 44, 46]. Authors of 1 paper reported on the prevalence of anti-Purkinje cell antibodies “similar to those found in paraneoplastic cerebellar degeneration”, but did not specify if tests for well-characterized onconeural antibodies were performed [39]. Two studies investigated the prevalence of well-characterized onconeural antibodies in the CSF of patients with psychotic and depressive syndromes [42, 45]. Thirteen papers included data on clinical characteristics of patients with a particular paraneoplastic neurological syndrome and/or positive onconeural antibody test result [25–28, 30–32, 35–38, 47, 49]. The authors of 4 studies reported on clinical characteristics of autoimmune and paraneoplastic limbic encephalitis in general, including cases associated with onconeural antibodies [2, 34, 40, 48]. Using the QUADAS-2, we found that 18 of 27 studies had a high applicability concern regarding patient selection (see Table 3 and Fig. 2) [2, 25–28, 30–38, 40, 47–49]. These studies did not evaluate patients with psychiatric symptoms (our primary review question) but with paraneoplastic neurological syndromes or malignancies (secondary review questions). In addition, the same studies lacked a reference standard regarding psychiatric symptoms. This explains the high applicability concern and risk of bias for reference standards in these 18 studies (Table 3).

### Patient population

The 27 studies included in the review had heterogeneous patient populations (total *n* = 3820). Nine studies included patients with psychiatric disorders (total *n* = 3066) [17, 29, 39, 41–46]. The remaining studies described symptoms from patients with a particular paraneoplastic neurological syndrome and/or positivity to a particular onconeural antibody (*n* = 555) [25–28, 30–33, 35–38, 47, 49], or autoimmune/limbic encephalitis (*n* = 199) [2, 34, 40, 48]. Due to the heterogeneity in patient populations (Table 4) a meta-analysis was considered inappropriate.

### Onconeural antibody testing

The majority of studies employed immunoblot or western blot techniques as the reference standard for detecting onconeuronal antibodies [2, 17, 26–28, 30–37, 40, 42–47], often in combination with indirect immunoflourocence (IIF) or immunhistochemistry [2, 17, 26–28, 30–34, 37, 40, 43, 44, 46, 48, 49]. When immunoblot/western blot was not used, IIF was performed [25, 29, 38, 39, 41]. There was a large variability in the specific antibodies analyzed and number of antibodies analyzed in each study. Authors from 2 studies did not use specific antibody testing but analyzed Purkinje cell antibodies in general [25, 39].

### Reference standard

Six studies used a validated reference standard for judgment of psychiatric disorders (ICD-10, DSM-III-R or DSM-IV) [17, 29, 39, 41, 43, 46]. Three other studies also included patients with psychiatric disorders [42, 44, 45]. These studies were conducted in secondary or tertiary psychiatric departments and it is thus likely that a board-certified psychiatrist evaluated the patients although this was not clearly stated. However, the studies were judged to have an unclear bias and applicability concern due to lack of information on the reference standard used. Hansen et al. screened for depressive symptoms using BDI (Becks Depression Inventory) and review of patient charts, but did not assess other psychiatric symptoms [48]. The remaining 17 studies did not have a reference standard for the evaluation of psychiatric symptoms. These studies were not designed to screen for psychiatric symptoms and/or disorders [2, 25–28, 30–38, 40, 47, 49].

### Serum prevalence studies

Six studies reported on the prevalence of 3 or more well-characterized onconeural antibodies in patients with psychiatric disorders [17, 29, 41, 43, 44, 46]. Dahm et al. screened patients with schizophrenia (*n* = 1378), affective disorders (*n* = 310), borderline personality disorders (*n* = 42) and healthy blood donors (*n* = 1703) for the presence of a wide range of onconeural antibodies (all as listed in this review protocol, except anti-SOX1). There were no differences in prevalence of any of the onconeural antibodies in healthy individuals and in patients from any of the diagnostic groups [41]. Sæther et al. screened 585 patients admitted to acute psychiatric inpatient care for the presence of all antibodies in this review protocol, except anti-Ma1 [46]. Only 1 patient tested positive (anti-recoverin). Kruse et al. examined serum from 213 and CSF from 10 psychiatric inpatients for a broad spectrum of antibodies including anti-Hu, -Ri, -Tr, -SOX1, -Yo, -CRMP-5 and -Amphiphysin [44]. One patient suffering from frontotemporal dementia tested positive to anti-CRMP-5. The remaining 212 patients and 173 healthy controls tested negative to all the above-mentioned antibodies. Haukanes et al. screened 169 adult ADHD patients for the presence of anti-Yo, -Hu, -Ri, -Ma2, -CRMP-5 and -Amphiphysin [43]. While 10 of the patients had antibodies targeting Purkinje cells, none tested positive to well-characterized onconeural antibodies. Black et al. did not find anti-Yo, -Hu, -Ri or -Amphiphysin among 13 patients diagnosed with obsessive-compulsive disorder [29]. Laadhar et al. reported unspecified antineuronal nuclear autoantibodies (ANNA) in 20/103 patients with schizophrenia, schizoaffective and bipolar disorder compared to 0/41 healthy controls. However, only 2 of them had well-characterized onconeural antibodies (anti-Ri). Further they reported antibodies directed to the cytoplasm of Purkinje cells in 6/103 patients and 2/41 controls. Three of the patients and none of the controls were positive to a well-characterized onconeural antibody (anti-Yo) [17]. Laadhar et al. only performed tests for 3 well-characterized onconeural antibodies (Anti-Hu, anti-Ri, and anti-Yo).

Another prevalence study screened 48 patients with psychiatric disorders (24 schizophrenia, 17 bipolar disorder and 7 obsessive-compulsive disorder) and 52 healthy controls for anti-Purkinje cell antibodies “similar to those found in paraneoplastic cerebellar degeneration”. It was not reported whether tests for well-characterized onconeural antibodies associated with paraneoplastic cerebellar degeneration (i.e. anti-Yo) were performed. Eleven of 48 psychiatric patients had anti-Purkinje antibodies compared to none of the controls [39]. In this study, Purkinje cell antibodies were associated with acute clinical presentations and positive psychopathological symptoms (hallucinations, delusions, bizarre behavior and thought disturbances) [39]. However, according to the QUADAS-2, applicability concerns regarding the index test in this study were high because information on specific testing for well-characterized onconeural antibodies was lacking.

### Cerebrospinal fluid studies in patients with psychiatric disorders

Endres et al. reported on the prevalence of intracellular onconeural antibodies in the cerebrospinal fluid (CSF) of patients with psychotic and depressive syndromes [42, 45]. Among 180 patients with psychotic syndromes, CSF from 142 patients were screened for the presence of anti-Yo, -Hu, -Ri, -CRMP-5/CV2, -Ma1, -Ma2, -SOX1 and -Amphiphysin [42]. Three patients tested positive to anti-Yo, 1 to anti-Hu and 1 to anti-CRMP-5/CV2. All these patients had schizophreniform or schizoaffective syndromes. Examining patients with depressive syndromes, the same authors found CSF from 63 patients to be negative for the above-mentioned intracellular antibodies [45]. None of the CSF studies included a control group.

### Studies on autoimmune encephalitis

In a study on 103 patients with encephalitis of possibly autoimmune origin, 9 patients tested positive for a well-characterized onconeural antibody (7 anti-Ri, 2 anti-Yo) [40]. Of these, 2 anti-Ri positive patients presented with the combination of psychosis and seizures, whereas 1 anti-Yo positive patients presented with behavioral changes. In another study, 30 of 50 patients with paraneoplastic limbic encephalitis tested positive to well-characterized onconeural antibodies (18 anti-Hu, 10 anti-Ma1/Ma2, 2 anti-Ma1) [2]. Psychiatric symptoms were present in 21/50 patients and included hallucinations and changes in affect and personality. The prevalence of psychiatric symptoms in patients testing positive to anti-Hu (5/18) or anti-Ma1/Ma2 (6/10) was not different from that in other patients (10/22). Yet another study investigated the clinical, magnetic resonance imaging and electroencephalographic findings in 24 patients with paraneoplastic limbic encephalitis [34]. The authors reported well-characterized onconeural antibodies in 10 patients (6 anti-CRMP5 and 4 anti-Hu). All patients tested negative for anti-Ri, anti-Yo and anti-Tr. Psychiatric symptoms were noted in 12 of the 24 patients with paraneoplastic limbic encephalitis, including depression, anxiety, personality changes and hallucinations. Seven patients had psychiatric symptoms as their initial manifestation. Eight out of 10 patients with anti-CRMP-5 or anti-Hu antibodies had clinical or radiological evidence of extralimbic encephalitis involvement compared to 4 out of 12 patients without these antibodies (*p* = 0.04). However, the authors did neither provide data on the degree of limbic involvement nor of the psychiatric symptomatology in these two groups. Hansen et al. aimed to assess the efficacy of immunotherapy in patients with different types of limbic encephalitis [48]. In their paper, they also provided clinical characteristics of 11 patients with onconeural antibodies (anti-Ma2, -Yo, -SOX1, -Recoverin or -Amphiphysin) and compared them to that of 11 anti-GAD65 positive patients. The authors found that patients with onconeural antibodies had a significantly greater improvement of depression scores (Becks Depression Inventory) following immunotherapy as compared to anti-GAD65-positive patients. Other psychiatric symptoms, however, were not assessed.

### Studies on individual onconeural antibodies

*Anti-Hu* was the subject of investigation in 4 papers. Dalmau et al. presented a clinical study including 71 patients with anti-Hu associated paraneoplastic encephalomyelitis/sensory neuronopathy [26]. They described 15 patients (21%) presenting with limbic symptoms such as confusion, depression, anxiety and memory loss. Six of these patients presented with partial-complex seizures that included gustatory, auditory, or olfactory hallucinations. In another study 16 patients with limbic encephalitis and small cell lung cancer were included [28]. Psychiatric symptoms such as depression, anxiety, personality changes, and hallucinations were frequent in both anti-Hu positive (7/8) and negative (7/8) patients. Following neurological stabilization in 73 anti-Hu positive patients with paraneoplastic encephalomyelitis/sensory neuronopathy, 2 patients were found to have depressive symptoms [33]. Aiming to differentiate anti-Hu positive patients with peripheral neuropathy and encephalitis by CSF parameters, the authors of another paper noted that some of the patients with limbic involvement presented with anxiety and aggressive behavior [49]. However, no systematic evaluation of psychiatric symptoms was performed.

Four papers described clinical symptoms associated to *anti-Ma1 and/or anti-Ma2.* In the initial description of the anti-Ma2 antibody, Voltz et al. referred to 10 patients with testicular cancer, paraneoplastic limbic or brainstem encephalitis, and the presence of serum anti-Ma2 antibodies [31]. To further assess the symptoms of anti-Ma2-associated encephalitis, Dalmau et al. reported a comprehensive clinical analysis of 38 patients, including the 10 mentioned previously [35]. The presenting features included psychiatric symptoms such as “nervous breakdowns” (2 patients), loss of self-confidence (1 patient) and panic attacks (1 patient). In another study of 22 cases of anti-Ma1/Ma2 associated paraneoplastic neurological syndromes, 2 patients presented with psychiatric symptoms (personality change, obsessive compulsive symptoms) [37]. Investigating hypocretin-1 CSF levels in patients with anti-Ma2 associated encephalitis; Overeem et al. reported that 3 out of 6 patients had psychiatric symptoms (depression, personality changes) [36]. However, these patients also developed neurological symptoms such as seizures and diplopia.

*Anti-Yo* associated paraneoplastic neurological syndromes often present with cerebellar ataxia. In a clinical analysis by Peterson et al. of 55 patients with *anti-Yo* antibodies and paraneoplastic cerebellar degeneration 10 patients had cognitive impairment, including emotional lability and memory deficits [27]. Other psychiatric symptoms were not recorded. McKeon et al. described the clinical profiles of 83 patients testing positive to anti-Yo at the Mayo Clinic during a 21-year period. From clinical records, they noted personality changes in 4 patients [38]. Hammack et al. compared patients with paraneoplastic cerebellar degeneration with Purkinje cell antibodies with patients without. The authors did not state if tests for well-characterized onconeural antibodies associated to paraneoplastic cerebellar degeneration (i.e. anti-Yo) were performed. Thus, this study was subject to significant applicability concerns regarding the index tests. The authors reported that mental status was abnormal more frequently in antibody positive (10/16) than in antibody negative patients (5/16). Depression or psychosis were present in 6 of 16 seropositive patients compared to 3 of 16 seronegative patients, whereas the remaining patients with altered mental status had dementia or delirium [25].

In 116 patients with *CRMP-5/CV2* antibodies, psychiatric abnormalities were reported in 24 patients (11 personality changes, 9 depression, 4 psychosis) [32]. It is unclear how many of these patients had co-occurring neurological symptoms, however.

Two papers focusing on *anti-amphiphysin* met the inclusion criteria. One of them reported five cases of anti-amphiphysin associated paraneoplastic neurological syndromes [30]. In 1 of these patients the initial symptoms were anxiety and depression. Later, the patient’s neurological status deteriorated and he developed olfactory and auditory hallucinations. The authors of another study described clinical manifestations and immunotherapy response of patients with non-stiff anti-amphiphysin syndrome [47]. Four out of 20 patients were described as irritable, but all of them had co-existing symptoms such as cognitive impairment or seizures. Another patient had been treated for psychotic symptoms in a psychiatric department for 10 years before amphiphysin antibodies were discovered. This patient additionally suffered from seizures, cognitive impairment, depressive symptoms and emotional lability. The authors noted that the psychiatric symptoms improved following treatment with IVIG (intravenous immunoglobulin) and oral Prednisolone.

## Discussion

Our primary research question was whether onconeural antibody status predicts the psychopathological profile in patients with psychiatric symptoms. Based on the six studies designed to answer this question [17, 29, 41, 43, 44, 46], it seems fair to conclude that the serum prevalence of well-characterized onconeural antibodies in patients with psychiatric disorders is generally low. Indeed, the prevalence does not seem to be significantly different from that in healthy controls. However, these studies include patients fulfilling the criteria for primary psychiatric disorders. It could be hypothesized that patients with psychiatric symptoms admitted to medical or surgical facilities because of a co-occurring somatic disease might have a higher prevalence.

CSF studies are rare in patients with psychiatric disorders; hence, the data on antibody prevalence in CSF are even more limited. Interestingly, Endres et al. found CSF from five of 142 patients with psychotic syndromes to be positive for anti-Yo, −Hu or -CRMP-5/CV2 [42].

To advance this field there is a need for collaboration between psychiatrist and neuroimmunologists. Future studies investigating the significance of onconeural antibodies for psychiatric symptomatology should not only assess the prevalence of these antibodies in psychiatric diagnostic categories. They should also investigate associations to symptom severity (e.g. degree of hallucinations, delusions and depression) and symptom domains not captured be the classical disorders (e.g. agitation and symptom fluctuation). Further, longitudinal studies are needed to establish whether a positive onconeural antibody test result in patients with psychiatric symptoms is a trait (stable over time) or state (associated with exacerbations of psychiatric symptoms).

One of our two secondary review questions was about onconeural antibody status possibly predicting the psychopathological profile in patients with malignancies. Our literature search yielded no studies designed to answer this question. The other secondary research questions concerned the possibility that onconeural antibody status might predict the psychopathological profile in patients with paraneoplastic CNS syndromes. Many of the included studies evaluated the clinical phenotypes of syndromes associated to a particular onconeural antibody. However, comparisons of symptoms in patients with a given syndrome (e.g. limbic encephalitis) with and without onconeural antibodies were only presented in three studies. Gultekin et al. compared clinical characteristics in three groups of patients with paraneoplastic limbic encephalitis (patients positive to anti-Hu, anti-Ma2 and others) [2]. Similarly, Alamowitch et al. compared clinical characteristics of patients with limbic encephalitis and small cell lung cancer with and without anti-Hu antibodies [28]. Both studies reported similar prevalence of psychiatric symptoms in patients with and without onconeural antibodies. However, the studies were small and included only a few of the relevant onconeural antibodies. Hansen et al. reported depressive symptoms in 73% of LE patients with onconeural antibodies as compared to 50% in LE patients with GAD65 antibodies [48]. Surprisingly, the authors reported that the depression scores improved significantly more in onconeural antibody positive patients as compared to the anti-GAD65 group following immunotherapy.

Our review has a few limitations. Only six prevalence studies of well-characterized onconeural antibodies in patients with psychiatric disorders were identified [17, 29, 41, 43, 44, 46]. These studies generally had low a low risk of bias as assessed by QUADAS-2. However, the studies differed in inclusion criteria and setting, and some of them did not include all antibodies described in the review protocol. The largest study used indirect immunofluorescence to screen for onconeural antibodies [41], but did not perform a second confirmatory test (e.g. immunoblot or ELISA) which recommended in some guidelines [7]. Eighteen of the studies designed to answer one of our secondary research questions were subject to a high risk of bias regarding the reference standard [2, 25–28, 30–38, 40, 47–49]. This was mainly due to the lack of a structured psychiatric evaluation and of validated diagnostic tools. The same studies also had high applicability concerns with regards to patient selection and reference standards.

## Conclusions

Although psychiatric symptoms do occur in patients with malignancies and paraneoplastic CNS syndromes, the few available studies suggest that well-characterized onconeural antibodies are infrequent in patients with psychiatric disorders per se.

## Additional file

Additional file 1: What is the significance of onconeural antibodies for psychiatric symptomatology? A systematic review. (DOCX 96 kb)
