# Supplementary material for: What is the significance of onconeural antibodies for psychiatric symptomatology? A systematic review
Source: BMC Psychiatry. 2017 May 3;17:161. doi: 10.1186/s12888-017-1325-z (PMC5415831; doi:10.1186/s12888-017-1325-z)
Supplement: Additional file 1: — What is the significance of onconeural antibodies for psychiatric symptomatology? A systematic review. (DOCX 96 kb) [file 12888_2017_1325_MOESM1_ESM.docx]

**Additional file 1:**

**What is the significance of onconeural antibodies for psychiatric symptomatology? A systematic review**

Onconeural Antibodies in Psychiatry –

a systematic review and meta-analysis

(Protocol from January 2015)

Sverre Georg Sæther1-2, Morten Schou1-2,

Arne Vaaler1-2, Solveig Reitan1-2 and Daniel Kondziella2-3

1St. Olav’s University Hospital, Department of Psychiatry, Trondheim, Norway

2Inst. of Neuroscience, Norwegian University of Science and Technology, Trondheim, Norway

3Departments of Neurology and Clinical Neurophysiology, Rigshospitalet, Copenhagen University Hospital, Copenhagen, Denmark

Corresponding author:

Sverre Georg Sæther

Department of Psychiatry, Østmarka

Trondheim University Hospital

P.b 3008, Lade, 7441 Trondheim

sverrege@gmail.com

1. B A C K G R O U N D

Paraneoplastic neurological syndromes, including paraneoplastic limbic encephalitits, associated with onconeural antibodies can present with psychiatric symptoms. The present review investigates the evidence for the association of onconeuronal antibodies with the occurrence, maintenance and worsening of psychiatric symptoms in patients with (and without) paraneoplastic disease~~s~~.

Onconeural antibodies target intracellular antigens in tumors and neuroectodermal tissues~~,~~ and are associated with various types of cancer and clinical syndromes. Well-characterized onconeural antibodies are anti-Hu (ANNA-1), Ri (ANNA-2), Yo, CRMP5 (CV2), Ma1, Ma2 (Ta), Amphiphysin, Recoverin, Tr and SOX1.^1,2^ Although useful biomarkers of paraneoplastic neurological syndromes, it is likely that these antibodies represent an epiphenomenon and that neuronal injury is mediated by cytotoxic T-cell immunity^3-5^.

Paraneoplastic limbic encephalititis typically evolves over days to weeks and includes memory disturbances, seizures and psychiatric symptoms such as irritability, hallucinations, depression, and personality disturbances^6,7^. Hypersomnia and delusional thought content have been described as well ^8,9^. In a recent report, the prevalence of onconeural antibodies was of the same magnitude in healthy individuals and patients with schizophrenia, affective disorders and personality disorders^10^, e.g. anti-amphiphysin (2.2%), anti-Yo (0.6%), anti-Ma2 (0.5%) and anti-Ma1 (0.5%).

Patients with autoimmune disorders associated with onconeural antibodies may present with psychiatric symptoms as the only clinical feature^7,11,12^, suggesting that onconeuronal antibodies still play a role in susceptible individuals. This systematic review and meta-analysis aims at determining the evidence for the association of onconeuronal antibodies with the occurrence, maintenance and worsening of psychiatric symptoms in patients with (and without) paraneoplastic diseases.

- 1. Target condition being diagnosed

The target condition is psychiatric symptoms in patients with serum onconeural antibodies. We will address:

1. Affective symptoms (lowered vs raised/irritable mood)
2. Consciousness (decreased level vs hyperalertness including hypersomnia/insomnia)
3. Psychosis (including negative symptoms, hallucinations, delusions, disturbances of thought and/or behavior)
4. Cognition (global confusion vs focal neuropsychological defects)
5. Personality changes (e.g. obsessive-compulsive behavior vs disinhibition)

We will not address symptoms likely to be of neurological/somatic origin, such as focal neurological deficits (e.g sensory loss, paresis, apraxia, aphasia), seizures, hyperthermia, endocrine dysfunction.

- 1. Index test

The index test comprises onconeural antibodies in patient serum or cerebrospinal fluid. We will address the following well-characterized onconeural antibodies”^1,2^: anti-Hu (ANNA-1), Ri (ANNA-2), Yo, CRMP5 (CV2), Ma1, Ma2 (Ta), Amphiphysin, Recoverin, Tr and SOX1 irrespective of the time of analysis (e.g. early or late in the course of the disease, single or repeated assessments).

- 1. Clinical pathway

Typically, onconeural antibodies are tested in patients with a clinical suspicion of a paraneoplastic neurological syndrome as part of a routine clinical work-up. However, analysis of these antibodies may also be performed as part of screening procedures (for either research or clinical purposes) in cancer and psychiatric patients, (i.e.in patients without a suspected paraneoplastic disorder).

- 1. Rationale

Patients with paraneoplastic disorders with onconeural antibodies frequently present with psychiatric symptoms, sometimes as the only clinical feature^7,11,12^. Nevertheless, discussion in the psychiatric literature is sparse^13-15^. We therefore plan a systematic review of the literature in order to analyze the existing data on the association of onconeuronal antibodies with the occurrence, maintenance and worsening of psychiatric symptoms in patients with (and without) paraneoplastic diseases.

2. O B J E C T I V E S

2.1. Primary objective

The main objective is to examine the association of onconeuronal antibodies with the occurrence, maintenance and worsening of psychiatric symptoms in patients with (and without) paraneoplastic diseases. Using the PICO approach, we phrase the following primary research question: In patients with psychiatric symptoms (Population), does a positive onconeural antibody titer (Intervention) compared to a negative titer (Comparison) predict a different psychopathological profile, i.e. a greater burden of affective, cognitive and/or psychotic symptoms (Outcome)?

2.2. Secondary objectives

Secondary objectives include the following:
1) In patients with malignancies (P), does a positive onconeural antibody titer (I) compared to a negative titer (C) predict the presence and/or degree of psychiatric symptoms, i.e. a greater burden of affective, cognitive and/or psychotic symptoms (O)?

2) In patients with a verified or suspected paraneoplastic neurological syndrome (P), does a positive onconeural antibody titer (I) compared to a negative titer (C) predict the presence and/or degree of psychiatric symptoms, i.e. a greater burden of affective, cognitive and/or psychotic symptoms (O)?

2.3. Investigation of sources of heterogeneity

We will attempt to explore possible sources of heterogeneity. These will likely be related to variances in methods of antibody analysis and the sample population examined.

As to antibody analysis, the gold standard is the characteristic immunohistochemical staining pattern on brain tissue sections combined with immunoblotting using recombinant purified proteins. However, analytic methods differ considerably between published studies^16-18^. We will include all analytic methods (e.g immunofluorescense, immunoprecipitation techniques, immunohistochemistry, immunoblot). Data on the specificity and sensitivity of the tests will be recorded when available. Studies using non-validated tests will be excluded.

As to study populations, some papers may focus exclusively on patients with psychiatric disorders, others on patients with malignancies, paraneoplastic neurological syndromes, or simply a positive antibody positive titer.

The extracted information will be tabulated and analyzed for possible heterogeneity with respect to definitions, inclusion criteria, techniques and other methods. As outlined below (3.3.6.) we will analyze the identified studies for possible publication bias using a funnel plot if deemed appropriate. If the selected studies do not state the precise methods used for analysis of antibodies and/or the composition of the population sample, we will try to contact the corresponding authors for further information.

3. M E T H O D S

3.1. Criteria for considering studies for this review

3.1.1. Types of studies

We will evaluate all retrospective, prospective, observational and/or interventional studies (as detailed below) describing the prevalence of onconeural antibodies and/or symptomatology of onconeural antibody positive patients in the above mentioned population samples. If a metaanalysis is appropriate, we will include all studies providing data on the frequency of psychiatric symptoms in the respective population samples. In order to reduce the influence of convenience samples, we will not consider studies with n ≤ 10. We will include studies published in all languages if a reliable translation into English is possible. We will exclude articles that concern patients already used in another article by the same authors (or the same institution) unless the methods sections make it clear that the patients do not overlap.

3.1.2. Participants

Patients aged 16 or older presenting within or outside institutionalized care. We will include patients irrespective of the severity of their disease or co-morbidities and irrespective of the timing of antibody analysis, e.g before or after cancer treatment.

3.1.3. Index tests

See above (1.2.).

3.1.4. Target conditions

See above (1.1.).

3.1.5. Reference standards

We will consider as reference standards the established clinical criteria for malignancies, paraneoplastic syndromes and psychiatric disorders according to standard clinical criteria, e.g. ICD-10, DSM-3-R and DSM-4.

3.2. Search methods for identification of studies

3.2.1. Electronic searches

We will search the following databases for relevant English literature from January 1, 1980 to December 31, 2014 (this search will be updated shortly before submission of the manuscript in order to include also the newest references): Cochrane Central Register of Controlled Trials (The Cochrane Library), Medline (PubMed), EMBASE, and clinicaltrials.gov. We will use the following search terms: (((paraneoplastic OR onconeural OR onconeuronal OR malignancy OR cancer)) AND (psychiatry OR depression OR major depression OR mood OR affective OR hallucinations OR psychosis OR cognition OR dementia OR confusion OR mania OR irritability)) AND (antibody OR antibodies). Non-English literature will be included if an English Abstract is available and a reliable translation of the manuscript into English possible. Reports exclusively dealing with data on pediatric patients (age below 16 years) will not be included. The references of relevant articles will be manually searched to identify additional articles. Further, papers will be cross-referenced using the ‘cited by’ function on PubMed. If necessary, personal communication with authors will be attempted via email or phone in order to obtain additional relevant data. The complete search strategies (including MeSH headings for searches in PubMed) will be saved and recorded in an appendix.

3.3. Data collection and analysis

3.3.1. Selection of studies

A comprehensive literature search will be performed without language restriction (other than specified in 3.1.2.) in order to identify relevant studies for this review. The search will be limited from January 1980 onwards as onconeural antibodies have not been well-described before that date. Titles will be reviewed first, followed by evaluation of the abstracts with titles suggesting that a study might be of relevance. Then eligible studies will be identified on the basis of their full text. The initial selection will be performed by one author (SGS), whereas quality assessment will be done blind by two assessors. Thus, all potentially relevant articles (as listed in 3.1.1.) will be reviewed and graded according to the quality and level of evidence by SGS, using QUADAS-2^19^, and confirmed for inclusion by a second author (see 3.3.3.). We will use proprietary reference manager software to manage the large number of studies, and we will document the study selection in a detailed flow chart.

3.3.2. Data extraction and management

Following identification of relevant studies, one of the authors (SGS) will extract the relevant information from each study, which will be double-checked by a second author. In addition to the information listed in the Methods section we will record 1) journal name and Vancouver-style reference, 2) study design (e.g. systematic review, cross-sectional study), 3) method of recruitment (e.g. prospective or retrospective), 4) study setting, 5) characteristics of the patient population (e.g. age, gender, co-morbidities). This information will be stored in a dedicated database. This review will be reported following the PRISMA criteria^20^.

3.3.3. Assessment of methodological quality

Using the Quality Assessment of Diagnostic Accuracy Studies-2 (QUADAS-2), a recent modified version of QUADAS^19^, two of the authors will independently assess the methodological quality of each included study, as outlined above. The QUADAS-2 comprises four domains: (1) participant selection, (2) index test, (3) reference standard, and (4) flow of participants through the study and timing of the index tests and reference standard (flow and timing). Each domain is assessed for risk of bias, and the first three domains are also assessed for concerns regarding applicability. Risk of bias and concerns about applicability are judged as “low”, “high” or “unclear”. (For assessment of possible reporting bias see 3.3.6.) We will resolve disagreement between the two reviewing authors by consensus. If this is not possible, a third author will make the final decision (DK).

3.3.4. Statistical analysis and data synthesis

Depending on the results of the literature search and review, we will propose to conduct a meta-analysis on all available numerical data which report on 1) the frequency of onconeural antibodies in patients with psychiatric symptoms and/or diagnoses, 2) frequency of psychiatric symptoms in patients with onconeural antibodies, and 3) the psychopathological profile of patients with onconeural antibodies. This will be subject to the quality of the studies, study design, risk of bias and the clinical case for combination.

3.3.5. Investigations of heterogeneity

See above (2.2.).

3.3.6. Assessment of reporting bias

In order to address possible publication bias or exaggeration of treatment effects in small studies of low quality we will analyze the identified studies using a funnel plot (scatter plot of the treatment effects estimated from individual studies on the horizontal axis against a measure of study size on the vertical axis^21^, if appropriate.

4. R E F E R E N C E S

1. Graus F, Delattre JY, Antoine JC, et al. Recommended diagnostic criteria for paraneoplastic neurological syndromes. *Journal of neurology, neurosurgery, and psychiatry* 2004; **75**(8): 1135-40.

2. Stich O, Rauer S. [Paraneoplastic neurological syndromes and autoimmune encephalitis]. *Der Nervenarzt* 2014; **85**(4): 485-98; quiz 99-501.

3. Bernal F, Graus F, Pifarre A, Saiz A, Benyahia B, Ribalta T. Immunohistochemical analysis of anti-Hu-associated paraneoplastic encephalomyelitis. *Acta neuropathologica* 2002; **103**(5): 509-15.

4. Voltz R, Dalmau J, Posner JB, Rosenfeld MR. T-cell receptor analysis in anti-Hu associated paraneoplastic encephalomyelitis. *Neurology* 1998; **51**(4): 1146-50.

5. Benyahia B, Liblau R, Merle-Beral H, Tourani JM, Dalmau J, Delattre JY. Cell-mediated autoimmunity in paraneoplastic neurological syndromes with anti-Hu antibodies. *Annals of neurology* 1999; **45**(2): 162-7.

6. Didelot A, Honnorat J. Paraneoplastic disorders of the central and peripheral nervous systems. *Handbook of clinical neurology* 2014; **121**: 1159-79.

7. Gultekin SH, Rosenfeld MR, Voltz R, Eichen J, Posner JB, Dalmau J. Paraneoplastic limbic encephalitis: neurological symptoms, immunological findings and tumour association in 50 patients. *Brain : a journal of neurology* 2000; **123 ( Pt 7)**: 1481-94.

8. Rojas-Marcos I, Graus F, Sanz G, Robledo A, Diaz-Espejo C. Hypersomnia as presenting symptom of anti-Ma2-associated encephalitis: case study. *Neuro-oncology* 2007; **9**(1): 75-7.

9. Koide R, Shimizu T, Koike K, Dalmau J. EFA6A-like antibodies in paraneoplastic encephalitis associated with immature ovarian teratoma: a case report. *Journal of neuro-oncology* 2007; **81**(1): 71-4.

10. Dahm L, Ott C, Steiner J, et al. Seroprevalence of autoantibodies against brain antigens in health and disease. *Annals of neurology* 2014; **76**(1): 82-94.

11. Bosemani T, Huisman TA, Poretti A. Anti-Ma2-associated paraneoplastic encephalitis in a male adolescent with mediastinal seminoma. *Pediatric neurology* 2014; **50**(4): 433-4.

12. Matsumoto L, Yamamoto T, Higashihara M, et al. Severe hypokinesis caused by paraneoplastic anti-Ma2 encephalitis associated with bilateral intratubular germ-cell neoplasm of the testes. *Movement disorders : official journal of the Movement Disorder Society* 2007; **22**(5): 728-31.

13. Newman NJ, Bell IR, McKee AC. Paraneoplastic limbic encephalitis: neuropsychiatric presentation. *Biological psychiatry* 1990; **27**(5): 529-42.

14. Cornelius JR, Soloff PH, Miewald BK. Behavioral manifestations of paraneoplastic encephalopathy. *Biological psychiatry* 1986; **21**(7): 686-90.

15. Kayser MS, Kohler CG, Dalmau J. Psychiatric manifestations of paraneoplastic disorders. *The American journal of psychiatry* 2010; **167**(9): 1039-50.

16. Johannis W, Renno JH, Wielckens K, Voltz R. Ma2 antibodies: an evaluation of commercially available detection methods. *Clinical laboratory* 2011; **57**(5-6): 321-6.

17. Monstad SE, Vedeler CA. An immunoprecipitation assay for the detection of onconeural antibodies. *Acta neurologica Scandinavica Supplementum* 2006; **183**: 71-2.

18. Maat P, Brouwer E, Hulsenboom E, et al. Multiplex serology of paraneoplastic antineuronal antibodies. *Journal of immunological methods* 2013; **391**(1-2): 125-32.

19. Whiting PF, Rutjes AW, Westwood ME, et al. QUADAS-2: a revised tool for the quality assessment of diagnostic accuracy studies. *Annals of internal medicine* 2011; **155**(8): 529-36.

20. Liberati A, Altman DG, Tetzlaff J, et al. The PRISMA statement for reporting systematic reviews and meta-analyses of studies that evaluate healthcare interventions: explanation and elaboration. *BMJ (Clinical research ed)* 2009; **339**: b2700.

21. Egger M, Davey Smith G, Schneider M, Minder C. Bias in meta-analysis detected by a simple, graphical test. *BMJ (Clinical research ed)* 1997; **315**(7109): 629-34.

**SEARCH STRATEGY**

EMBASE:

1. hu antibody/ or hu antigen/ (817)

2. exp paraneoplastic syndrome/ or paraneoplastic syndrome*.mp. (13541)

3. (paraneoplastic or onconeural or onconeuronal or onconeuro*).mp. (16936)

4. 1 or 2 or 3 (17586)

5. exp mental disease/ (1920905)

6. ("mental disorder" or "mental disorders" or "mental disease" or "mental diseases" or mental or psychiatry or depression or major depression or mood or affective or hallucinations or psychosis or cognition or dementia or confusion or mania or irritability).mp. (1634739)

7. 5 or 6 (2493001)

8. 4 and 7 (2802)

9. exp neoplasm/ (3984188)

10. exp carcinoma/ (931395)

11. ("neoplasms" or "neoplasm" or "malignancy" or "cancer" or "cancers" or carcinoma*).mp. (3416993)

12. 9 or 10 or 11 (4447558)

13. 7 and 12 (167001)

14. (antibody or antibodies).mp. (1287398)

15. 13 and 14 (7434)

16. 1 or 8 or 15 (9557)

17. (animal not human).mp. (3953077)

18. limit 16 to (adult <18 to 64 years> or aged <65+ years>) (3370)

19. 18 not 17 (3351)

PUBMED:

(((((("Hu Paraneoplastic Encephalomyelitis Antigens"[Mesh] OR "Paraneoplastic Syndromes"[Mesh] OR paraneoplastic OR onconeural OR onconeuronal OR onconeuro*) AND ("Psychiatry and Psychology Category"[Mesh] OR "Mental Disorders"[Mesh] OR "mental disorder" OR "mental disorders" OR "mental disease" OR "mental diseases" OR mental OR psychiatry OR depression OR major depression OR mood OR affective OR hallucinations OR psychosis OR cognition OR dementia OR confusion OR mania OR irritability))) OR (("neoplasms"[MeSH] OR "neoplasms" OR "neoplasm" OR "malignancy" OR "cancer" OR "cancers" OR "carcinoma"[MeSH Terms] OR carcinoma*) AND ("Mental Disorders"[Mesh] OR "mental disorder" OR "mental disorders" OR "mental disease" OR "mental diseases" OR psychiatry OR depression OR major depression OR mood OR affective OR hallucinations OR psychosis OR cognition OR dementia OR confusion OR mania OR irritability) AND (antibody OR antibodies))) NOT medline[sb])) OR ((((("neoplasms"[MeSH] OR "neoplasms" OR "neoplasm" OR "malignancy" OR "cancer" OR "cancers" OR "carcinoma"[MeSH Terms] OR carcinoma*) AND ("Mental Disorders"[Mesh] OR "mental disorder" OR "mental disorders" OR "mental disease" OR "mental diseases" OR psychiatry OR depression OR major depression OR mood OR affective OR hallucinations OR psychosis OR cognition OR dementia OR confusion OR mania OR irritability) AND (antibody OR antibodies))) NOT (("Animals"[Mesh] NOT "Humans"[Mesh]))) AND adult[MeSH]) (2244)

COCHRANE:

ID                  Search           Hits

#1                  MeSH descriptor: [Hu Paraneoplastic Encephalomyelitis Antigens] explode all trees  (3)

#2                 MeSH descriptor: [Paraneoplastic Syndromes] explode all trees           (62)

#3                 paraneoplastic or onconeural or onconeuronal or onconeuro*                 (77)

#4                 #1 or #2 or #3                   (114)

#5                 MeSH descriptor: [Neoplasms] explode all trees             (60559)

#6                 MeSH descriptor: [Carcinoma] explode all trees              (10137)

#7                 neoplasms or neoplasm or malignan* or cancer or cancers or carcinoma*                     125355

#8                 #5 or #6 or #7                   (129151)

#9                 MeSH descriptor: [Mental Disorders] explode all trees   (51617)

#10                mental or mental disorder or mental disorders or mental disease or mental diseases or psychiatry or depression or major depression or mood or affective or hallucinations or psychosis or cognition or dementia or confusion or mania or irritability         (112828)

#11                #9 or #10     (130847)

#12                #8 and #11    (7466)

#13                antibody or antibodies         (26075)

#14                #12 and #13 (354)

Clinicaltrials.gov:

**("neoplasms" OR "neoplasm" OR "malignancy" OR "cancer" OR "cancers" OR carcinoma) AND (antibody OR antibodies) AND (mental OR depression OR irritability OR Anxiety) | Adult, Senior** (49)

and

**(paraneoplastic OR onconeural OR onconeuronal OR onconeuro*)** (age 18+) **(**71)

**Papers excluded after full-text eligibility assessment (with reasons for exclusion)**

No screening or information on psychiatric symptomatology (n=22)

A., L., A.F., C., J., H., P., S.-S., A., T., Y., B., … Graus, F. (2006). Hu-antibody-positive patients with or without cancer have similar clinical profiles [5]. *Journal of Neurology, Neurosurgery and Psychiatry*, *77*(8), 996–997. ELEC. http://doi.org/10.1136/jnnp.2005.085753

A., S., M., R., R., V., B., G., F., G., W., G., & J., H. (2016). Prostate cancer, Hu antibodies and paraneoplastic neurological syndromes. *Journal of Neurology*. ELEC, A. Storstein, Department of Neurology, Haukeland University Hospital, Bergen, Norway. E-mail: astt@helse-bergen.no: Dr. Dietrich Steinkopff Verlag GmbH and Co. KG. Retrieved from http://ovidsp.ovid.com/ovidweb.cgi?T=JS&PAGE=reference&D=emed14&NEWS=N&AN=20160245021

A., S., S.E., M., M., H., K., M., D., V., E., L., … C., V. (2011). Onconeural antibodies: Improved detection and clinical correlations. *Journal of Neuroimmunology*. ELEC, A. Storstein, Department of Neurology, Haukeland University Hospital, Bergen, Norway. E-mail: anette.storstein@helse-bergen.no: Elsevier (P.O. Box 211, Amsterdam 1000 AE, Netherlands). Retrieved from http://ovidsp.ovid.com/ovidweb.cgi?T=JS&PAGE=reference&D=emed10&NEWS=N&AN=2011125433

B., B., P., B., R., D., T., H., & S., R. (2015). “Non-classical” paraneoplastic neurological syndromes associated with well-characterized antineuronal antibodies as compared to “classical” syndromes - More frequent than expected. *Journal of the Neurological Sciences*. ELEC, B. Berger, University Hospital Freiburg, Department of Neurology and Neurophysiology, Breisacher Strasse 64, Freiburg D-79106, Germany: Elsevier. Retrieved from http://www.elsevier.com/locate/jns

Candler, P. M., Hart, P. E., Barnett, M., Weil, R., & Rees, J. H. (2004). A follow up study of patients with paraneoplastic neurological disease in the United Kingdom. *J Neurol Neurosurg Psychiatry*, *75*(10), 1411–1415. Journal Article. http://doi.org/10.1136/jnnp.2003.025171

Elrington, G. M., Murray, N. M., Spiro, S. G., & Newsom-Davis, J. (1991). Neurological paraneoplastic syndromes in patients with small cell lung cancer. A prospective survey of 150 patients. *J Neurol Neurosurg Psychiatry*, *54*(9), 764–767. Journal Article.

G., G., A.G., K., Z., L., B., B.-H., T., L., A., C., … Członkowska, A. Positivity of serum “classical” onconeural antibodies in a series of 2063 consecutive patients with suspicion of paraneoplastic neurological syndrome, 259Journal of Neuroimmunology 75–80 (2013). ELEC, G. Gromadzka, Institute of Psychiatry and Neurology, Second Department of Neurology, Sobieskiego 9, 02-957 Warsaw, Poland. E-mail: gragrom@ipin.edu.pl: Elsevier (P.O. Box 211, Amsterdam 1000 AE, Netherlands). http://doi.org/10.1016/j.jneuroim.2013.04.007

Honnorat, J., Antoine, J. C., Derrington, E., Aguera, M., & Belin, M. F. (1996). Antibodies to a subpopulation of glial cells and a 66 kDa developmental protein in patients with paraneoplastic neurological syndromes. *J Neurol Neurosurg Psychiatry*, *61*(3), 270–278. Journal Article.

Honnorat, J., Cartalat-Carel, S., Ricard, D., Camdessanche, J. P., Carpentier, A. F., Rogemond, V., … Antoine, J. C. (2009). Onco-neural antibodies and tumour type determine survival and neurological symptoms in paraneoplastic neurological syndromes with Hu or CV2/CRMP5 antibodies. *J Neurol Neurosurg Psychiatry*, *80*(4), 412–416. Journal Article. <http://doi.org/10.1136/jnnp.2007.138016>

O., S., E., K., P., B., S., J., C., R., R., V., & S., R. (2012). SOX1 antibodies in sera from patients with paraneoplastic neurological syndromes. *Acta Neurologica Scandinavica*. ELEC, O. Stich, Department of Neurology, Albert Ludwigs University Freiburg, Breisacher Strasse 64, D-79106 Freiburg, Germany. E-mail: oliver.stich@uniklinik-freiburg.de: Blackwell Publishing Ltd (9600 Garsington Road, Oxford OX4 2XG, United Kingdom). Retrieved from http://ovidsp.ovid.com/ovidweb.cgi?T=JS&PAGE=reference&D=emed10&NEWS=N&AN=2012197732

P., S.-S., T., P., H., G.-B., W., K., & S., M. (2013). Neurological paraneoplastic syndromes in lung cancer patients. (P. M., Ed.)*Respiratory Regulation - The Molecular Approach*. ELEC, S. Michalak, Department of Neurochemistry and Neuropathology, University of Medical Sciences, 49 Przybyszewskiego St., Poznan 60-355, Poland. E-mail: swami622@gmail.com: Springer New York (233 Spring Street, New York NY 10013-1578, United States). Retrieved from http://ovidsp.ovid.com/ovidweb.cgi?T=JS&PAGE=reference&D=emed10&NEWS=N&AN=2012606827

Pieret, F., Sindic, C. J., Chalon, M. P., Warny, M., Bolyn, S., Dehaene, I., … C., L. (1996). The anti-Hu syndrome: a clinical and immunological study of 7 cases. *Acta Neurologica Belgica*, *96*(2), 108–116. ELEC. Retrieved from http://ovidsp.ovid.com/ovidweb.cgi?T=JS&PAGE=reference&D=emed4&NEWS=N&AN=8711983

Psimaras, D., Carpentier, A. F., & Rossi, C. (2010). Cerebrospinal fluid study in paraneoplastic syndromes. *J Neurol Neurosurg Psychiatry*, *81*(1), 42–45. Journal Article. http://doi.org/10.1136/jnnp.2008.159483

S., S., J., G., B., D. L., M., V. den B., H., H., B., V. der H., … P., S. S. (2003). Paraneoplastic cerebellar degeneration associated with antineuronal antibodies: Analysis of 50 patients. *Brain*. ELEC, P. Sillevis Smitt, Department of Neurology, Erasmus MC, Dr Molewaterplein 40, 3015 GD Rotterdam, Netherlands. E-mail: p.sillevissmitt@erasmusmc.nl: Oxford University Press. Retrieved from http://ovidsp.ovid.com/ovidweb.cgi?T=JS&PAGE=reference&D=emed6&NEWS=N&AN=2003221036

S.J., O., Y., G., E.J., D., P., K., & G.C., C. (2005). Anti-Hu antibody neuropathy: A clinical, electrophysiological, and pathological study. *Clinical Neurophysiology*. ELEC, S.J. Oh, Department of Neurology, University of Alabama at Birmingham, Veterans Affairs Medical Center, Birmingham, AL 35294, United States. E-mail: shinjoh@uab.edu: Elsevier Ireland Ltd. Retrieved from http://ovidsp.ovid.com/ovidweb.cgi?T=JS&PAGE=reference&D=emed7&NEWS=N&AN=2005085412

Saiz, A., Bruna, J., Stourac, P., Vigliani, M. C., Giometto, B., Grisold, W., … F., G. (2009). Anti-Hu-associated brainstem encephalitis. *Journal of Neurology, Neurosurgery and Psychiatry*, *80*(4), 404–407. ELEC. http://doi.org/10.1136/jnnp.2008.158246

Saiz, A., Dalmau, J., Butler, M. H., Chen, Q., Delattre, J. Y., De Camilli, P., & Graus, F. (1999). Anti-amphiphysin I antibodies in patients with paraneoplastic neurological disorders associated with small cell lung carcinoma. *J Neurol Neurosurg Psychiatry*, *66*(2), 214–217. Journal Article.

Keime-Guibert, F., Graus, F., Fleury, A., Rene, R., Honnorat, J., Broet, P., & Delattre, J. Y. (2000). Treatment of paraneoplastic neurological syndromes with antineuronal antibodies (Anti-Hu, anti-Yo) with a combination of immunoglobulins, cyclophosphamide, and methylprednisolone. *J Neurol Neurosurg Psychiatry*, *68*(4), 479–482. Journal Article.

M., R., C., V., & A., S. (2015). Paraneoplastic neurological syndromes in lung cancer patients with or without onconeural antibodies. *Journal of the Neurological Sciences*. ELEC, M. Raspotnig, University of Bergen, Department of Clinical Medicine, Jonas Lies veg 87, Bergen 5021, Norway: Elsevier. Retrieved from www.elsevier.com/locate/jns

M., T., P., S., O., G., T., G., N., K., C., P., … F., B. (2010). Anti-SOX1 antibodies in patients with paraneoplastic and non-paraneoplastic neuropathy. *Journal of Neuroimmunology*. ELEC, M. Tschernatsch, Dept. of Neurology, Justus-Liebig-University, Am Steg 14, 35385 Giessen, Germany. E-mail: Marlene.Tschernatsch@neuro.med.uni-giessen.de: Elsevier (P.O. Box 211, Amsterdam 1000 AE, Netherlands). Retrieved from http://ovidsp.ovid.com/ovidweb.cgi?T=JS&PAGE=reference&D=emed9&NEWS=N&AN=2010484597

M.A., K., Y., S., M.S., U., S.A., J., R., M. K., C., S., & D., R. (2015). Incidence and spectrum of paraneoplastic neurological syndromes: single center study. *Journal of Neuro-Oncology*. ELEC, M.A. Kanikannan, Department of Neurology, Nizam’s Institute of Medical Sciences, Punjagutta, Hyderabad 500082, India: Springer New York LLC. Retrieved from http://www.wkap.nl/journalhome.htm/0167-594X

Keime-Guibert, F., Graus, F., Broet, P., Rene, R., Molinuevo, J. L., Ascaso, C., & Delattre, J. Y. (1999). Clinical outcome of patients with anti-Hu-associated encephalomyelitis after treatment of the tumor. *Neurology*, *53*(8), 1719–1723. Journal Article.

Study population not matching review questions (n=1)

J.-I., B., S.-T., L., K.-H., J., J.-S., S., J., M., T.-J., K., … Lee S.K.  AO  - Kim, K. J. O. http://orcid. org/000.-0002-0849-125X. (2016). Prevalence of antineuronal antibodies in patients with encephalopathy of unknown etiology: Data from a nationwide registry in Korea. *Journal of Neuroimmunology*. ELEC, K. Chu, Department of Neurology, Seoul National University Hospital, 101, Daehak-Ro Jongno-Gu, Seoul 03080, South Korea. E-mail: stemcell.snu@gmail.com: Elsevier. Retrieved from <http://www.elsevier.com/locate/jneuroim>

Case reports (n=5)

J.M., S., K., H., J., B., A.I., D. R., A., V., & M.N., R. (2003). Amnesia, cerebral atrophy, and autoimmunity. *Lancet*. ELEC, M.N. Rossor, Dementia Research Group, Institute of Neurology, University College, London WC1N 3BG, United Kingdom. E-mail: m.rossor@dementia.ion.ucl.ac.uk: Elsevier Limited. Retrieved from http://ovidsp.ovid.com/ovidweb.cgi?T=JS&PAGE=reference&D=emed6&NEWS=N&AN=2003156344

J.R., C., P.H., S., & B.K., M. (1986). Behavioral manifestations of paraneoplastic encephalopathy. *Biological Psychiatry*. ELEC, Western Psychiatric Institute and Clinic, Department of Psychiatry, University of Pittsburgh, School of Medicine, Pittsburgh, PA 15213 United States. Retrieved from http://ovidsp.ovid.com/ovidweb.cgi?T=JS&PAGE=reference&D=emed1b&NEWS=N&AN=1986212010

L.D.A., D., A.C., K., W.O., R., & J.M.M., G. (2002). Anti-amphiphysin associated limbic encephalitis: A paraneoplastic presentation of small-cell lung carcinoma [2]. *Journal of Neurology*. ELEC, L.D.A. Dorresteijn, Dept. of Neurology, University Medical Center St Radboud, Renier Postlaan 4, 6525 GC Nijmegen, Netherlands: Dr. Dietrich Steinkopff Verlag GmbH and Co. KG. Retrieved from http://ovidsp.ovid.com/ovidweb.cgi?T=JS&PAGE=reference&D=emed5&NEWS=N&AN=2002331056

Murphy, S. M., Khan, U., Alifrangis, C., Hazell, S., Hrouda, D., Blake, J., … Reilly, M. M. (2012). Anti Ma2-associated myeloradiculopathy: expanding the phenotype of anti-Ma2 associated paraneoplastic syndromes. *J Neurol Neurosurg Psychiatry*, *83*(2), 232–233. Journal Article. http://doi.org/10.1136/jnnp.2010.223271

Scheid, R., Voltz, R., Guthke, T., Bauer, J., Sammler, D., & von Cramon, D. Y. (2003). Neuropsychiatric findings in anti-Ma2-positive paraneoplastic limbic encephalitis. *Neurology*, *61*(8), 1159–1161. Journal Article.

Studies on other antibodies (n=3)

V., B., T., A., M.J., T., S., M., & J., D. (2015). Autoimmune encephalitis in postpartum psychosis. *American Journal of Psychiatry*. ELEC, V. Bergink: American Psychiatric Association. Retrieved from http://ajp.psychiatryonline.org/doi/pdf/10.1176/appi.ajp.2015.14101332

Zang, W., Zhang, Z., Feng, L., & Zhang, A. (2016). The clinical diagnosis and treatment about 22 cases of limbic encephalitis were retrospectively analyzed. *Pakistan Journal of Pharmaceutical Sciences*, *29*(2 Suppl), 653–5. Retrieved from http://www.ncbi.nlm.nih.gov/pubmed/27113304

Z.O., A., D., K., Ayas, Z. O., Kotan, D., & Aras, Y. G. (2016). Autoimmune neurological syndromes associated limbic encephalitis and paraneoplastic cerebellar degeneration. *Neurosci Lett*, *632*, 187–191. ELEC. http://doi.org/10.1016/j.neulet.2016.08.038

**QUADAS-2**

**Phase 1: Review Question:**

In patients with psychiatric symptoms (Population), does a positive onconeural antibody titer (Intervention) compared to a negative titer (Comparison) predict a different psychopathological profile, i.e. a greater burden of affective, cognitive and/or psychotic symptoms (Outcome)?

Patients: Patients with psychiatric symptoms.

Index test: Onconeural antibody

Reference standard: ICD 10, DSM IV, DSM-IIIR, expert opinion by board-certified psychiatrist

Target condition: Psychopathological profile (psychiatric symptomatology)

**Phase 2: Review specific tailoring**

DOMAIN 1: PATIENT SELECTION

Risk of bias: Could the selection of patients have introduced bias?

Signalling questions:

1. Was a consecutive sample of patients enrolled? Yes/No/Unclear
2. Did the study avoid inappropriate exclusions? Yes/No/Unclear
3. Was a retrospective design avoided? Yes/No/Unclear

Applicability: Are there concerns that the included patients and setting do not match the review question; are there concerns that the included patients have not been evaluated for psychiatric symptoms?

DOMAIN 2: INDEX TEST

Risk of bias: Could the conduct or interpretation of the index test have introduced bias?

Signalling questions:

1. Were the index test results (antibody analysis) interpreted without knowledge of the psychopathological profile of the individual patient? Yes/No/Unclear
2. Were the antibody analysis done by a validated method? Yes/No/Unclear

Applicability: Are there concerns that the index test (onconeural antibody analysis), its conduct, or interpretation, differ from the review question? Yes/No/Unclear

DOMAIN 3: REFERENCE STANDARD

Risk of bias: Could the reference standard (ICD 10/DSM IV, DSM-III-R or expert opinion by a board-certified psychiatrist), its conduct, or its interpretation, have introduced bias?

Signalling questions:

1. Did the authors specify psychiatric symptoms as defined by ICD-10, DSM-IV, DSM-III-R or according to expert opinion by a board-certified psychiatrist? Yes/No/Unclear
2. Were the reference standard results interpreted without knowledge of the results of the index test? Were psychiatric symptoms classified before patients were known to be antibody positive? Yes/No/Unclear

Applicability: Are there concerns that the target condition (psychopathological profile) has not been defined using the reference standard (ICD 10, DSM IV, DSM-III-R, expert opinion by a board-certified psychiatrist)?

DOMAIN 4: FLOW AND TIMING

Risk of bias: Could the patient flow have introduced bias?

Signalling questions:

1. Was there an appropriate interval between index test (onconeural antibody analysis) and reference standard (evaluation of psychiatric symptoms) (within an arbitrarily defined time period of three months)? Yes/No/Unclear
2. Did all patients receive the same reference standard (evaluation of psychiatric symptoms)? Yes/No/Unclear
3. Where all patients with antibody positive status screened for psychiatric symptoms? Yes/No/Unclear

**Support for judgment for QUADAS-2:**

**1) Anti-Hu-Associated PEM/PSN: A Clinical Study of 71 Patients. Dalmau et al**

Patient Selection*: Low risk of bias*: A consecutive sample. No inappropriate exclusions. Retrospective design. *High applicability concern*: the patients do not match the primary review question.

Index test: *Low risk of bias*: Validated method. *Low applicability concern*: the index test matches the primary review question.

Reference standard: *High risk of bias*: No systematic assessment of psychiatric symptoms. *High applicability concern*: no defined reference standard for psychiatric symptoms.

Flow and timing: *High risk of bias*: No information on evaluation of psychiatric symptoms.

**2) Purkinje Cell Cytoplasmic Autoantibody Type 1 Accompaniments. McKeon et al**

Patient Selection*: Low risk of bias*: A consecutive sample. No inappropriate exclusions. Retrospective design. *High applicability concern*: the patients do not match the primary review question.

Index test: *Low risk of bias*: Validated method. *Low applicability concern*: the index test matches the primary review question.

Reference standard: *High risk of bias*: No screening of mental status. *High applicability concern*: no defined reference standard for psychiatric symptoms.

Flow and timing: *High risk of bias*: No reference standard on psychiatric symptoms/diagnoses. Unknown if all patients were screened for psychiatric symptoms.

**3) Paraneoplastic cerebellar degeneration: A clinical analysis of 55 anti-Yo… Peterson et al.**

Patient Selection: *Low risk of bias*: A consecutive sample. No inappropriate exclusions. Retrospective design. *High applicability concern*: the patients do not match the primary review question.

Index test: *Low risk of bias*. Validated method. *Low applicability concern*: the index test matches the primary review question.

Reference standard: *High risk of bias*: No screening of mental status. *High applicability concern*: no defined reference standard for psychiatric symptoms.

Flow and timing: *High risk of bias*: No reference standard on psychiatric symptoms/diagnoses. Unknown if all patients were screened for psychiatric symptoms.

**4) Seroprevalence of Autoantibodies against Brain Antigens in Health and Disease. Dahm et al.**

Patient Selection: *Unclear risk of bias*: Unclear if patients were included consecutively. Unclear if inappropriate exclusions were avoided. Cross-sectional design. *Low applicability concern*: the patients match the primary review question.

Index test: *Low risk of bias*. Validated method. *Low applicability concern*: the index test matches the primary review question.

Reference standard: *Low risk of bias*: Inclusion of GRAS cohort according to DSM-IV in specialist care. Unclear how the patients with affective disorders and borderline personality disorder were diagnosed. *Low applicability concern*: GRAS cohort using DSM-IV in the diagnostics.

Flow and timing: *Low risk of bias*: Appropriate time between index test and reference standard. Evaluation of psychiatric symptoms was performed in the patients with psychiatric disorders. Unclear if controls were evaluated for psychiatric symptoms.

**5) Clinical analysis of anti-Ma2-associated encephalitis. Dalmau et al.**

Patient Selection: *Low risk of bias*: Patients were included consecutively. Inappropriate exclusions were avoided. Retrospective design. *High applicability concern*: the patients do not match the primary review question.

Index test: *Low risk of bias*. Validated method. *Low applicability concern*: the index test matches the primary review question.

Reference standard: *High risk of bias*: No information on psychiatric assessment. *High applicability concern*: No defined reference standard for psychiatric symptoms/diagnoses.

Flow and timing: *High risk of bias*: Appropriate time between index test and reference standard. No reference standard on psychiatric symptoms/diagnoses. Unknown if all patients were screened for psychiatric symptoms.

**6) Anti-Ma and anti-Ta associated paraneoplastic neurological syndromes: 22 newly diagnosed patients and review of previous cases. Hoffmann et al.**

Patient Selection: *Unclear risk of bias*: Unclear if patients were included consecutively. Unclear if inappropriate exclusions were avoided. Retrospective design. *High applicability concern*: the patients do not match the primary review question.

Index test: *Low risk of bias*. Validated method. *Low applicability concern*: the index test matches the primary review question.

Reference standard: *High risk of bias*: No information on psychiatric assessment. *High applicability concern*: No defined reference standard for psychiatric symptoms/diagnoses.

Flow and timing: *High risk of bias*: No reference standard on psychiatric symptoms/diagnoses. Unknown if all patients were screened for psychiatric symptoms.

**7) CRMP-5 Neuronal Autoantibody: Marker of Lung Cancer and Thymoma-Related Autoimmunity. Yo et al.**

Patient Selection: *Low risk of bias*: Patients were included consecutively. Inappropriate exclusions were avoided. Retrospective design. *High applicability concern*: the patients do not match the primary review question.

Index test: *Low risk of bias*. Validated method. *Low applicability concern*: the index test matches the primary review question.

Reference standard: *High risk of bias*: No information on psychiatric assessment. *High applicability concern*: No defined reference standard for psychiatric symptoms/diagnoses.

Flow and timing: *High risk of bias*: No reference standard on psychiatric symptoms/diagnoses. Unknown if all patients were screened for psychiatric symptoms.

**8) Antiamphiphysin Antibodies Are associated With Various Paraneoplastic Neurological Syndromes and Tumors. Antoine et al.**

Patient Selection: *Low risk of bias*: Patients were included consecutively. Inappropriate exclusions were avoided. Retrospective design. *High applicability concern*: the patients do not match the primary review question.

Index test: *Low risk of bias*. Validated method. *Low applicability concern*: the index test matches the primary review question.

Reference standard: *High risk of bias*: No information on psychiatric assessment. *High applicability concern*: No defined reference standard for psychiatric symptoms/diagnoses.

Flow and timing: *High risk of bias*: No reference standard on psychiatric symptoms/diagnoses. Unknown if all patients were screened for psychiatric symptoms.

**9) Limbic encephalitis and small cell lung cancer. Clinical and immunological features. Alamowitch et al.**

Patient Selection: *Low risk of bias*: Patients were included consecutively. Inappropriate exclusions were avoided. Retrospective design. *High applicability concern*: the patients do not match the primary review question.

Index test: *Low risk of bias*. Validated method. *Low applicability concern*: the index test matches the primary review question.

Reference standard: *High risk of bias*: No information on psychiatric assessment. *High applicability concern*: No defined reference standard for psychiatric symptoms/diagnoses.

Flow and timing: *High risk of bias*: No reference standard on psychiatric symptoms/diagnoses. Unknown if all patients were screened for psychiatric symptoms.

**10) A serological marker of paraneoplastic limbic and brain-stem encephalitis in patients with testicular cancer. Voltz et al.**

Patient Selection: *Low risk of bias*: Patients were included consecutively. Inappropriate exclusions were avoided. Retrospective design. *High applicability concern*: the patients do not match the primary review question.

Index test: *Low risk of bias*. Validated method. *Low applicability concern*: the index test matches the primary review question.

Reference standard: *High risk of bias*: No information on psychiatric assessment. *High applicability concern*: No defined reference standard for psychiatric symptoms/diagnoses.

Flow and timing: *High risk of bias*: No reference standard on psychiatric symptoms/diagnoses. Unknown if all patients were screened for psychiatric symptoms.

**11) Hypocretin-1 CSF levels in anti-Ma2 associated encephalitis. Overeem et al.**

Patient Selection: *High risk of bias*: Patients were not included consecutively. Inappropriate exclusions seem to be avoided. Retrospective design. *High applicability concern*: the patients do not match the primary review question.

Index test: *Low risk of bias*. Validated method. *Low applicability concern*: the index test matches the primary review question.

Reference standard: *High risk of bias*: No information on psychiatric assessment. *High applicability concern*: No defined reference standard for psychiatric symptoms/diagnoses.

Flow and timing: *High risk of bias*: No reference standard on psychiatric symptoms/diagnoses. Unknown if all patients were screened for psychiatric symptoms.

**12) Paraneoplastic limbic encephalitis: neurological symptoms, immunological findings and tumor association in 50 patients. Gultekin et al.**

Patient Selection: *Unclear risk of bias*: Unclear if patients were included consecutively. Unclear if inappropriate exclusions were avoided. Retrospective design. *High applicability concern*: the patients do not match the primary review question.

Index test: *Low risk of bias*. Validated method. *Low applicability concern*: the index test matches the primary review question.

Reference standard: *High risk of bias*: No information on psychiatric assessment. *High applicability concern*: No defined reference standard for psychiatric symptoms/diagnoses.

Flow and timing: *High risk of bias*: No reference standard on psychiatric symptoms/diagnoses. Unknown if all patients were screened for psychiatric symptoms.

**13) Paraneoplastic Cerebellar Degeneration: A Clinical Comparison of Patients With and Without Purkinje Cell Cytoplasmic Antibodies. Hammack et al.**

Patient Selection: *Unclear risk of bias*: Unclear if patients were included consecutively. Unclear if inappropriate exclusions were avoided. Retrospective design. *High applicability concern*: the patients do not match the primary review question.

Index test: *Low risk of bias*. Validated method. *High applicability concern*: the index test does not measure well-characterized onconeural antibodies.

Reference standard: *High risk of bias*: No information on psychiatric assessment. *High applicability concern*: No defined reference standard for psychiatric symptoms/diagnoses.

Flow and timing: *High risk of bias*: No reference standard on psychiatric symptoms/diagnoses. Unknown if all patients were screened for psychiatric symptoms.

**14) Anti-Purkinje Cell and Natural Autoantibodies in a Group of Psychiatric Patients. Evidences for a Correlation with the Psychopathological Status. Delle Chiaie et al.**

Patient Selection: *High risk of bias*: Patients were not included consecutively. The study states excluded patients, but not inclusion criteria. It seems unclear how patients with other psychiatric disorders (e.g. unipolar depressions) were grouped. Cross-sectional design. *Low applicability concern*: the patients match the primary review question.

Index test: *Low risk of bias*. Validated method. *High applicability concern*: the index test does not measure well-characterized onconeural antibodies, but rather “anti-Purkinje cell antibodies” which are not well-characterized and not explicitly mentioned in the review protocol.

Reference standard: *Low risk of bias*: DSM-IV. *Low applicability concern*: DSM-IV.

Flow and timing: *Low risk of bias*: Appropriate time interval between index test. The same reference standard applied on all patients (DSM-IV).

**15) High Prevalence of Antineuronal Antibodies in Tunisian Psychiatric Inpatients. Laadhar et al.**

Patient Selection: *Low risk of bias*: Patients were included consecutively. Unclear if inappropriate exclusions were avoided. Cross-sectional design. *Low applicability concern*: the patients match the primary review question.

Index test: *Low risk of bias*. Validated method. *Low applicability concern*: the index test matches the primary review question.

Reference standard: *Low risk of bias*: DSM-IV. *Low applicability concern*: DSM-IV.

Flow and timing: *Low risk of bias*: Appropriate time between index test and reference standard. All patients with psychiatric symptoms received the same reference standard (DSM-IV).

**16) Autoimmune causes of encephalitis syndrome in Thailand: prospective study of 103 patients. Saraya et al.**

Patient Selection: *Low risk of bias*: Patients were included consecutively. Inappropriate exclusions were avoided. Prospective design. *High applicability concern*: the patients do not match the primary review question.

Index test: *Low risk of bias*. Validated method. *Low applicability concern*: the index test matches the primary review question.

Reference standard: *High risk of bias*: No information on psychiatric assessment. *High applicability concern*: No defined reference standard for psychiatric symptoms/diagnoses.

Flow and timing: *High risk of bias*: No reference standard on psychiatric symptoms/diagnoses. Unknown if all patients were screened for psychiatric symptoms.

**17) Paraneoplastic syndrome-associated neuronal antibodies in adult ADHD. Haukanes et al.**

Patient Selection: *High risk of bias*: Patients were not included consecutively. Inappropriate exclusions were probably not avoided. Cross-sectional design. *Low applicability concern*: the patients match the primary review question.

Index test: *Low risk of bias*. Validated method. *Low applicability concern*: the index test matches the primary review question.

Reference standard: *Low risk of bias*: DSM-IV. *Low applicability concern*: DSM-IV.

Flow and timing: *Low risk of bias*: Appropriate time between index test and reference standard. All patients with psychiatric symptoms received the same reference standard (DSM-IV).

**18) Onconeural antibodies in acute psychiatric inpatient care. Sæther et al.**

Patient Selection: *Low risk of bias*: Patients were included consecutively. Avoided inappropriate exclusions. Cross-sectional design. *Low applicability concern*: the patients match the primary review question.

Index test: *Low risk of bias*. Validated method. *Low applicability concern*: the index test matches the primary review question.

Reference standard: *Low risk of bias*: ICD-10. *Low applicability concern*: ICD-10.

Flow and timing: *Low risk of bias*: Appropriate time between index test and reference standard. All patients with psychiatric symptoms received the same reference standard (ICD-10).

**19) Serological survey of adult patients with obsessive-compulsive disorder for neuron-specific and other autoantibodies. Black et al.**

Patient Selection: *Unclear risk of bias*: Unclear if patients were included consecutively and if inappropriate exclusions were avoided. Cross-sectional design. *Low applicability concern*: the patients match the primary review question.

Index test: *Low risk of bias*. Validated method. *Low applicability concern*: the index test matches the primary review question.

Reference standard: *Low risk of bias*: DSM-III-R. *Low applicability concern*: DSM-III-R.

Flow and timing: *Low risk of bias*: Appropriate time between index test and reference standard. All patients with psychiatric symptoms received the same reference standard (DSM-III-R).

**20) Psychiatric autoimmunity: N-methyl-d-Aspartate Receptor IgG and beyond. Kruse et al.**

Patient Selection: *High risk of bias*: Patients were not included consecutively. Unclear if inappropriate exclusions were made. Retrospective design. *Low applicability concern*: the patients match the primary review question.

Index test: *Low risk of bias*. Validated method. *Low applicability concern*: the index test matches the primary review question.

Reference standard: *Unclear risk of bias*: Study performed in psychiatric specialist care, but validated diagnostic method/instrument is not mentioned. *Unclear applicability concern*: Study performed in psychiatric specialist care, but validated diagnostic method/instrument is not mentioned.

Flow and timing: *Low risk of bias*: Appropriate time between index test and reference standard. All patients were assessed for psychiatric symptoms.

**21) Immunological findings in psychotic syndromes: a tertiary care hospital’s CSF sample of 180 patients. Endres et al 2015.**

Patient Selection: *High risk of bias*: Patients were included consecutively for one period, and not consecutively for another. Unclear if inappropriate exclusions were avoided. Cross-sectional design. *Low applicability concern*: the patients match the primary review question.

Index test: *Low risk of bias*. Validated method. *Low applicability concern*: the index test matches the primary review question.

Reference standard: *Unclear risk of bias*: Study performed in psychiatric specialist care, but validated diagnostic method/instrument is not mentioned. *Unclear applicability concern*: Study performed in psychiatric specialist care, but validated diagnostic method/instrument is not mentioned.

Flow and timing: *Low risk of bias*: Appropriate time between index test and reference standard. Evaluation of psychiatric symptoms was performed.

**22) Evidence of cerebrospinal fluid abnormalities in patients with depressive syndromes. Endres et al 2016.**

Patient Selection: *High risk of bias*: Patients were not included consecutively. Only patients with depressive syndromes and “potential organic signs” were included. Cross-sectional design. *Low applicability concern*: the patients match the primary review question.

Index test: *Low risk of bias*. Validated method. *Low applicability concern*: the index test matches the primary review question.

Reference standard: *Unclear risk of bias*: Study performed in psychiatric specialist care, but validated diagnostic method/instrument is not mentioned. *Unclear applicability concern*: Study performed in psychiatric specialist care, but validated diagnostic method/instrument is not mentioned.

Flow and timing: *Low risk of bias*: Appropriate time between index test and reference standard. Evaluation of psychiatric symptoms was performed.

**23) Survival and outcome in 73 anti-Hu positive patients with paraneoplastic encephalomyelitis/sensory neuronopathy. Sillevis Smitt et al.**

Patient Selection: *Low risk of bias*: A consecutive sample. No inappropriate exclusions. Retrospective design. *High applicability concern*: the patients do not match the primary review question.

Index test: *Low risk of bias*. Validated method. *Low applicability concern*: the index test matches the primary review question.

Reference standard: *High risk of bias*: No screening of mental status. *High applicability concern*: no defined reference standard for psychiatric symptoms.

Flow and timing: *High risk of bias*: No reference standard on psychiatric symptoms/diagnoses. Unknown if all patients were screened for psychiatric symptoms.

**24) Clinical, Magnetic Resonance Imaging, and Electroencephalographic Findings in Paraneoplastic Limbic Encephalitis. Lawn et al.**

Patient Selection: *High risk of bias*: Patients were not included consecutively. Inappropriate exclusions were probably not avoided. Retrospective design. *High applicability concern*: the patients do not match the primary review question.

Index test: *Low risk of bias*. Validated method. *Low applicability concern*: the index test matches the review question.

Reference standard: *High risk of bias*: No information on psychiatric assessment. *High applicability concern*: No defined reference standard for psychiatric symptoms/diagnoses.

Flow and timing: *High risk of bias*: No reference standard on psychiatric symptoms/diagnoses. Unknown if all patients were screened for psychiatric symptoms.

**25) Non-stiff anti-amphiphysin syndrome: Clinical manifestations and outcome after immunotherapy. Moon et al.**

Patient Selection: *Low risk of bias*: A consecutive sample. No inappropriate exclusions. Retrospective design. *High applicability concern*: the patients do not match the primary review question.

Index test: *Low risk of bias*. Validated method. *Low applicability concern*: the index test matches the primary review question.

Reference standard: *High risk of bias*: No screening of mental status. *High applicability concern*: no defined reference standard for psychiatric symptoms.

Flow and timing: *High risk of bias*: No reference standard on psychiatric symptoms/diagnoses. Unknown if all patients were screened for psychiatric symptoms.

**26) Seizure control and cognitive improvement via immunotherapy in late onset epilepsy patients with paraneoplastic versus GAD65 autoantibody-associated limbic encephalitis. Hansen et al.**

Patient Selection: *High risk of bias*: Not a consecutive sample. Inappropriate exclusions were not avoided. Retrospective design. *High applicability concern*: the patients do not match the primary review question.

Index test: *Low risk of bias*. Validated method. *Low applicability concern*: the index test matches the primary review question.

Reference standard: *High risk of bias*: No screening of mental status. *High applicability concern*: no defined reference standard for psychiatric symptoms.

Flow and timing: *High risk of bias*: Depressive symptoms were evaluated by BDI (Beck Depression Inventory) and chart review. However, other psychiatric symptoms were not evaluated systematically.

**27) Intrathecal synthesis of anti-Hu antibodies distinguishes patients with paraneoplastic peripheral neuropathy and encephalitis. Schwenkenbecher et al.**

Patient Selection: *Low risk of bias*: Consecutive sample. Inappropriate exclusions were avoided. Retrospective design. *High applicability concern*: the patients do not match the primary review question.

Index test: *Low risk of bias*. Validated method. *Low applicability concern*: the index test matches the primary review question.

Reference standard: *High risk of bias*: No screening of mental status. *High applicability concern*: no defined reference standard for psychiatric symptoms.

Flow and timing: *High risk of bias*: No reference standard on psychiatric symptoms/diagnoses. Unknown if all patients were screened for psychiatric symptoms.
